# α-Mangostin Induces Apoptosis and Inhibits Metastasis of Breast Cancer Cells via Regulating RXRα-AKT Signaling Pathway

**DOI:** 10.3389/fphar.2021.739658

**Published:** 2021-08-30

**Authors:** Xiuzhi Zhu, Jialin Li, Huiting Ning, Zhidong Yuan, Yue Zhong, Suzhen Wu, Jin-Zhang Zeng

**Affiliations:** ^1^ School of Pharmaceutical Sciences, Xiamen University, Xiamen, China; ^2^ School of Basic Medicine, Gannan Medical University, Ganzhou, China; ^3^ School of Pharmacy, Gannan Medical University, Ganzhou, China

**Keywords:** α-mangostin, apoptosis, RXRα/tRXRα, cyclin D1, breast cancer

## Abstract

Mangostin, which has the function of anti-inflammatory, antioxidant, and anticancer, etc, is one of the main active ingredients of the hull of the mangosteen. The main objective of the study was to elucidate its anti-cancer function and possible mechanism. α-Mangostin was separated and structurally confirmed. MTT method was used to check the effect of mangostin on breast cancer cell proliferation. Then the effect of α-Mangostin on the transcriptional activity of RXRα was tested by dual-luciferase reporter gene assay. And Western blot (WB) was used to detect the expression of apoptosis-related proteins or cell cycle-associated proteins after treatment. Also, this study was to observe the effects of α-Mangostin on the invasion of breast cancer cell line MDA-MB-231. α-Mangostin regulates the downstream effectors of the PI3K/AKT signaling pathway by degrading RXRα/tRXRα. α-Mangostin can trigger PARP cleavage and induce apoptosis, which may be related to the induction of upregulated BAX expression and downregulation of BAD and cleaved caspase-3 expression in MDA-MB-231 cells through blockade of AKT signaling. The experiments verify that α-Mangostin have evident inhibition effects of invasion and metastasis of MDA-MB-231 cells. Cyclin D1 was involved in the anticancer effects of α-Mangostin on the cell cycle in MDA-MB-231 cells. α-Mangostin induces apoptosis, suppresses the migration and invasion of breast cancer cells through the PI3K/AKT signaling pathway by targeting RXRα, and cyclin D1 has involved in this process.

## Introduction

Mangostin is a series of natural compounds isolated from the epicarp of the fruits of *Garcinia Mangostana Linn* which is one of the most popular herbal medicines (Nguyen et al., 2020). In traditional Chinese medicine, the fruit hull of mangosteen has been widely used in the treatment of diarrhea, diabetes, and cancer for centuries (Ittiudomrak et al., 2019; Zou et al., 2019). Also, the mangosteen fruit rind extracts have free radical scavenging and anti-acne activities (Phan et al., 2018). Since Mangostin was first isolated and identified in mangosteen fruit rind extracts (Zhang et al., 2017; Yeong et al., 2020), many biological activities and pharmacological studies of α-Mangostin have been performed in subsequent studies (Zhang et al., 2018). A suppression effect of α-Mangostin has been shown in breast cancer cells. α-Mangostin can indirectly induce the apoptosis of leukemia HL60 cells and human DLD-1 colon cancer cells through a reduction of mitochondrial transmembrane potential (Fu et al., 2018; Lee et al., 2018), and also inhibits the proliferation of DLD-1 cells in the G1 phase (Nakagawa et al., 2007). The cytotoxicity of more than 10 types of compounds extracted from the epicarp of the mangosteen fruit has been tested, of which α-Mangostin was the most toxic to human leukemia cell lines (Jasek et al., 2014; Austin et al., 2015). The toxicity of methanol extracts from mangosteen shells on human breast cancer SKBR3 cells was also confirmed (Scolamiero et al., 2018; Bissoli and Muscari, 2020), and the authors speculated that the active ingredient causes apoptosis by inhibiting low-density lipoprotein (LDL) oxidation and acid sheath phospholipase activity.

We have confirmed that α-Mangostin is highly toxic to human breast cancer SKBR3, MCF-7, and MDA-MB-231 cells. The experiments revealed that α-Mangostin can bind with RXRα in cells and is a good antagonist of this receptor. RXRα is a non-steroidal nuclear receptor, and the conformation of its ligand-binding domain (LBD) helix 12 (Chen et al., 2014) changes to regulate biological function when the corresponding ligand is present (Ishizawa et al., 2012). RXRα has a non-genetic function in the cytoplasm in addition to its transcriptional function in the nucleus (Chen et al., 2014). Recent studies have shown that RXRα can be truncated by restrictive hydrolysis in many tumor cells (Zheng et al., 2019), to produce RXRα with missing N-terminus, called tRXRα (truncated RXRα) (Zhou et al., 2010). Truncated RXRα is mainly localized to the cytoplasm, regulating downstream PI3K through interactions with p85, which activates the PI3K/AKT survival pathway and promotes the disorderly proliferation of tumor cells (Jiang et al., 2013).

The invasive metastasis of breast cancer is a complex evolutionary process with multiple factors and steps, regulated by the expression of multiple genes (Hamurcu et al., 2018). The breast cancer cells invasion is the main reason of recurrence after tumor resection (Fuste et al., 2016). So, chemotherapy is generally the main treatment for invasive breast cancer. Paclitaxel is commonly used for the treatment of invasive breast cancer since the 90s (Zhong et al., 2010). But this clinical drug has shortcomings as well, such as side effects, drug resistance, etc. So far, the treatment of triple negative breast cancer (TNBC) is still difficulty. There is an urgent need for suitable targeted drugs and more effective drugs for TNBC(Fuste et al., 2016).

In this study, we examined whether tRXRα serves as an intracellular target mediating the biological activities of α-Mangostin. Also, we investigated the mechanism by which α-Mangostin acts to promote tumor cell apoptosis and inhibit invasion and metastasis. Furthermore, we explored the possibility of dissociating the anticancer effects of α-Mangostin from its cell cycle inhibition activity.

## Materials and Methods

### Extraction and Separation

The fruits of Mangosteen were bought directly from the fruit market in Xiamen, China. The fruits were authenticated as *Garcinia Mangostana. Linn* by Professor Jin-zhang Zeng (School of pharmaceutical sciences and State Key Laboratory of Cellular Stress Biology, Xiamen University). The epicarp of the fruits (3 kg) was air-dried at room temperature and pulverized, then extracted with 95% ethanol (20 L) three times (36 h per time) at room temperature to yield an extract. The latter was concentrated in vacuo to yield a residue (504 g), which was suspended in H_2_O and partitioned with Ethyl acetate. The solvent was evaporated under reduced pressure to afford an Ethyl acetate extract (209 g). The Ethyl acetate extract was subjected to silica gel column chromatography (200–300 mesh) with a gradient eluant system of CH_2_CL_2_-CH_3_OH (80:1-40:1, v/v) to yield 8 fractions. TLC (thin-layer chromatography) was employed to monitor the process. Fraction 6 was subjected to silica gel column chromatography eluted with petroleum ether–Ethyl acetate (4:1–1:1, v/v) to afford 2 subfractions 6.1–6.2. Fraction 6.2 followed by semi-preparative HPLC with 94% ethanol to give compounds 1 (67 mg), 2 (72 mg), and 3 (36 mg). The chromatographic column used in this method is the capillary column of C-18ODS (20*250 mm).

### Structure Identification

The compounds were characterized by proton nuclear magnetic resonance, mass spectrometry, and elemental analysis. The compound 3 was identified as α-Mangostin according to the chromatographic behavior and mass spectral data by comparison with those of control. Spectral data: 1H-NMR (400 MHz,CDCl3):δ 1.62(6H,S, 19-CH_3_,20-CH_3_),1.73(3H,s, 14-CH3), 1.77(3H,s, 15-CH_3_), 3.20(2H,d,J = 6.8 Hz,H-11), 3.79(3H,S, 7-OCH_3_),4.09(2H,d,J = 6.8 Hz,H-16),5.16(2H,m,H-12, H-17),6.36(1H,S,H-4),6.80(1H,S,H-5),10.80(1H,brs,C-3-OH),13.78(1H,S,C-1-OH).13C-NMR (100MHz, CDCl_3_):δ 17.9(C-19), 18.2(C-14), 21.4(C-11), 25.8(C-15,20), 26.6(C-16), 62.0(7-OCH_3_), 93.3((C-4), 102.8(C-5),101.6(C-9a), 108.5(C-2), 112.2(C-8a), 121.5(C-12), 123.1(C-17), 133.1(C-13), 135.6(C-18), 137.0(C-8), 142.5(C-7), 154.5(C-4a), 155.3(C-10a), 155.8(C-6), 160.6(C-1), 161.6(C-3), 182.0(C-9).

### Cell Lines and Plasmids

The MCF-7, SKBR-3, MDA-MB-231, and HEK-293 T cells were obtained from the Type Culture Collection of the Chinese Academy of Sciences (Shanghai, China), and grown in DMEM (dulbecco’s modified eagle medium) supplemented with 10% fetal bovine serum (PAN Biotech, SA) at 37°C in an incubator containing 5% CO2. The pGAL4-RXRα-LBD plasmid was obtained by inserting the RXRα ligand-binding domain (LBD) cDNA sequence (amino acids 198–462) in-frame with the GAL4 DBD coding sequence in the pBind vector.

### CCND1 and RXRα siRNA

CCND1 and RXRα siRNA was purchased from DHARMACON and was transfected into cells using RNAi- MAX reagent.

### MTT Assay of Cell Viability and Proliferation

Three breast cancer cell lines were seeded at a density of 5–10 × 10^4^ cells/well in 96-well plates containing 100 μL medium (DMEM containing 10% FBS) for 18 h. Thereafter, the culture medium was removed and replaced with 100 μL medium (serum-free DMEM) containing various concentrations (40, 20, 10, 5, 2.5, and 1.2 μM) of α-Mangostin or 0.1% (v/v) dimethyl sulfoxide (DMSO) solvent only (control). The cells were grown for another 48 h. Subsequently, 10 μL MTT solution (5 g/L) was added to each well and the culture plates were incubated for 4 h to allow formazan formation. The culture medium was then removed, the formazan was solubilized by the addition of 150 μL DMSO, and the absorbance at 560 nm (A560) was measured with a microplate reader to calculate cell viability (%). The IC50(50% inhibitive concentration) value of the compound was calculated by Graphpad Prism 6.0.

### Apoptosis and Cell Cycle Analysis

The MDA-MB-231 cells were cultured at a density of 4 × 10^5^ cells/well in 6-well plates containing 1,600 μL medium for overnight culture. Then, the cells were treated with various concentrations of α-Mangostin, meanwhile, 5 μM concentration of paclitaxel acted as a control. The cells were harvested at the indicated times by trypsinization with 0.05% (w/v) trypsin in 0.5 mM ethylenediaminetetraacetic acid solution. The cells were then washed twice with cold phosphate-buffered saline (PBS), using centrifugation at 1,000 × g for 5 min at 4°C to harvest the cells each time. For apoptosis detection, the cell pellets were resuspended in 50 μL binding buffer (10 mM HEPES, pH 7.4, 140 mM NaCl, and 2.5 mM CaCl_2_) and stained with 5 μL annexin V-Alexa Fluor 488 and 5 μL PI for 30 min at room temperature in the dark. For the cell cycle study, the cell pellets were fixed in 200 μL cold 70% (v/v) ethanol at −20°C overnight, harvested, and washed as described above. The washed cell pellet was then suspended in 250 μL PBS containing 0.1 g/L RNase A and incubated at 37°C for 30 min. Thereafter, it was washed as described above, resuspended in staining buffer (12.5 μL PI (1 g/L in PBS)), and incubated at room temperature for 30 min in the dark. The samples were then analyzed by flow cytometry on an FC 500 MPL cytometer that recorded 10,000 events per sample. The experiment was performed in triplicate.

### Transient Transfection and Reporter Assays

293 T cells were grown in DMEM supplemented with 10% FBS. For dual-luciferase reporter assays, cells were seeded at a concentration of 5 × 10^4^ cells per well in 24-well plates and transfected with pGL5 luciferase reporter vector (40 ng/well) and pGAL4-RXRα-LBD expression vector (40 ng/well). Cells were then incubated with various concentrations of compounds for 12 h. Luciferase activities were measured using the Dual-Luciferase Assay System Kit.

### SPR (Surface Plasmon Resonance) Measurements

Binding experiments were carried out using Biacore S200 SPR sensors with control software version 3.0 and Sensor Chip CM5 (carboxymethylated dextran surface). All assays were carried out at 25°C. For the pre-binding experiment, pure RXRα-LBD protein was dissolved with acetate buffers with different pH values (pH4.0, pH4.5, pH5.0, and pH5.5) and flowed through the surface of the chip at a rate of 5 μL/min. Then, the chip surface was activated following a standard EDC/NHS protocol with Biacore PBS-EP buffer used as the running buffer. RXRα-LBD protein at a concentration of 0.4 g/L in 10 mM phosphate buffer pH 5.0 was then injected for 12 min followed by a 7- min injection of 1 M ethanolamine to inactivate residual active groups. Typically, approximately 2000 RU receptor protein was immobilized per flow cell. By reference to the coupling steps of RXRα-LBD protein, the protein was coupled to the chip. For the preparation of the α-Mangostin solution, the insoluble residue was pelleted by centrifugation and discarded. α-Mangostin was injected into the protein channel and blank channel at seven concentrations (0.625 nM, 1.25 nM, 2.5 nM, 5 nM, 10 nM, 20 nM, and 40 nM). The supernatant (200 μL) was injected at a flow rate of 20 μL/min. The protein binding period was set to 3 min, and the dissociation period was set to 300 s. The chip was regenerated with glycine-HCl (pH 2.5, 10 mM).

### Western Blotting

Cell lysates were prepared by lysing cells with lysis buffer (RIPA) containing 50 mM Tris-HCl, 150 mM NaCl, 1 mM EDTA, 0.1% SDS, 1% Na-deoxycholate, 1% Triton X-100, pH 7.4 with a cocktail of proteinase inhibitors on ice for 30 min. The cells were fixed with 80 μL/well cell lysates in 12-well plates. Equal amounts of the lysates were electrophoresed on 8% SDS-PAGE gels and transferred onto polyvinylidene difluoride membranes. The membranes were blocked with 5% nonfat milk in Tris-Buffered Saline and Tween 20 [50 mmol/L Tris–HCl (pH 7.4), 150 mmol/L NaCl, and 0.1% Tween 20] for 1 h, incubated with various primary antibodies for 48 h and detected with either anti-rabbit (1:5,000) or anti-mouse (1:5,000) secondary antibodies for 1 h. The final immunoreactive products were detected using enhanced chemiluminescence (ECL).

### Molecular Docking

The three-dimensional structure of human RXRα (PDB code: 3A9E) was downloaded from the Protein Data Bank. Protein preparation was performed by Chimera and autodock software and was saved as a locked PDB file for docking. Schrodinger software glide module was used to calculate the molecular docking of α-Mangostin and RXRα, list the possible binding sites, and determine the docking energy.

### Statistical Analyses

The quantitative data were obtained by three or more repeated experiments. Data were analyzed using an analysis of variance or Student’s t-test and were presented as the mean and standard error of the mean (±SEM).

## Results

### α-Mangostin Inhibits Proliferation and Induces Apoptosis of Breast Cancer Cells

**FIGURE 1:**
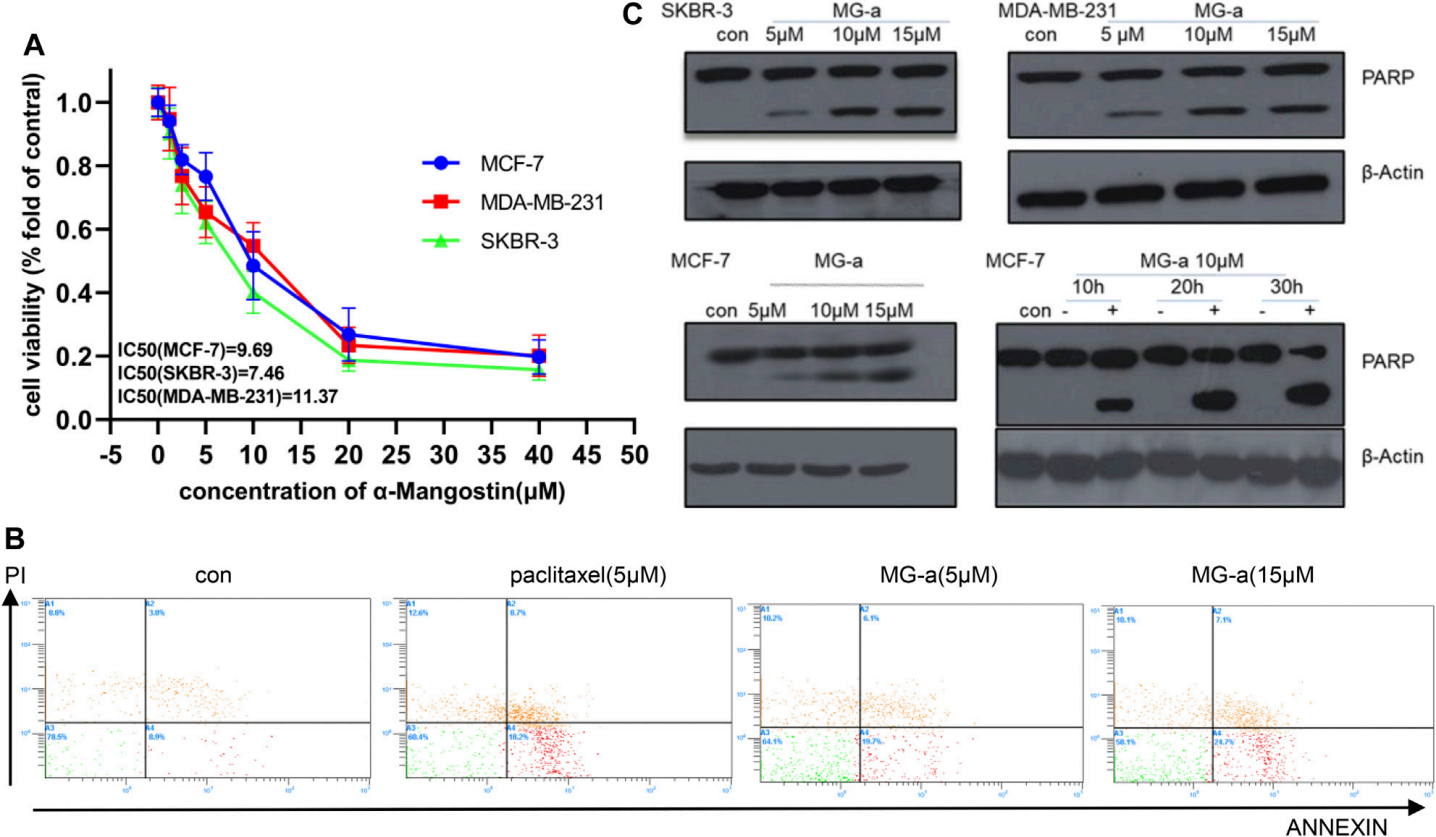
α-Mangostin (MG-a) inhibits proliferation and induces apoptosis of breast cancer cells. **(A)** α-Mangostin reduces breast cancer proliferation. **(B)** Apoptosis of MDA-MB-231cells treated with MG-a was assessed by flow cytometry. **(C)** PARP expression levels were determined by western blotting analysis in breast cancer cells after α-Mangostin treatment.

We first assessed the anti-proliferative effects of α-Mangostin in breast cancer cells. MCF-7, MDA-MB-231, and SKBR-3 cells were treated with increasing concentrations of α-Mangostin (0, 1.2, 2.5, 5, 10, 20, and 40 μM) for 48 h α-Mangostin significantly inhibited breast cancer cell proliferation in a dose-dependent manner. (Figure 1A). The IC50 values of α-Mangostin against MCF-7, MDA-MB-231, and SKBR-3 cells were 9.69, 11.37, and 7.46 μM, respectively. We used Annexin-V and PI double staining to detect the cytotoxicity of α-Mangostin by flow cytometry. MDA-MB-231 cells were treated with α-Mangostin using paclitaxel as a positive control. α-Mangostin could induce apoptosis of MDA-MB-231 cells in a concentration-dependent manner (Figure 1B).

To further verify the apoptotic effects of α-Mangostin in breast cancer cells at the molecular level, we detected the cleavage of PARP in MCF-7, SKBR-3, MDA-MB-231 cells by western blotting. The PARP protein in these cell lines had been significantly cleaved, and the cleavage increased with the increase in α-Mangostin concentration over 20 h treatment (Figure 1C). Meanwhile, the cleavage of PARP proteins in the MCF-7 cells increased in a time-dependent manner through treatment with 10 μM α-Mangostin.

### The Apoptotic Effects of α-Mangostin Depend on the PI3K/Akt Signaling Pathway

**FIGURE 2:**
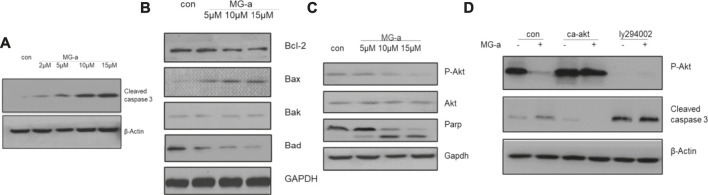
The apoptotic effects of α-mangostin depend on the PI3K/Akt signaling pathway. Cell apoptosis in MDA-MB-231 cells treated with α-Mangostin depends on the PI3K/Akt signaling pathway. **(A)** The expression levels of cleaved caspase-3 were determined by western blotting analysis in MDA-MB-231 cells after α-Mangostin treatment. **(B)** The expression levels of BCL-2 family proteins were determined by western blotting analysis in MDA-MB-231 cells after α-Mangostin treatment. **(C)** and **(D)**, The western blotting analysis demonstrated that PI3K/Akt signaling is required for the apoptosis of MDB-MD-231 cells induced by α-Mangostin.

Because of potential influence on suppressing the migration and invasion of TNBC Cells *in Vitro* by α-Mangostin, we chose MDA-MB-231 to perform further experiments. Western blotting was used to detect cleaved caspase-3 and BCL-2 protein family expression in MDA-MB-231 cells (Figure 2). Endogenous cleaved caspase-3 mostly related to BCL-2 protein family. The expression of BAD and BAX was affected by α-Mangostin. A graded decrease in BAD expression was detected following treatment of cells with the concentration gradient of α-Mangostin for 24 h, while BAX expression increased (Figure 2B).

The PI3K/Akt signaling pathway is one of the most important survival pathways in cells and has a very close relationship with tumor development (Zhang et al., 2018). We further explored whether α-Mangostin induces apoptosis through the PI3K/Akt signaling pathway. The expression of Akt was almost unchanged with the increase in α-Mangostin concentration, while the expression of P-Akt decreased in turn (Figure 2C). After treatment with 10 μM α-Mangostin, the expression of P-Akt (activated Akt) protein in cells was significantly reduced. P-Akt was significantly suppressed compared with that of the control group. To further verify whether the induction of apoptosis of breast cancer cells by α-Mangostin depends on the PI3K/Akt signaling pathway, we added PI3K/Akt inhibitor LY294002 (20 μM) to the medium of MDA-MB-231 cells for 6 h. Then the cells were treated with α-Mangostin for 20 h. The expression of P-Akt protein in cells was then detected. LY294002 inhibited the activation of Akt (Figure 2D) and enhanced the inhibitory effect of α-Mangostin on P-Akt. Our laboratory constructed a plasmid expressing continuously activated Akt (CA-Akt) by generating a point mutation in a critical region. We transfected the plasmid into MDA-MB-231 cells. After 24 h, the cells were treated with 10 μM α-Mangostin. We observed many apoptotic cells in the control group, while the apoptosis of MDA-MB-231 cells expressing CA-Akt was much less. Western blotting demonstrated the cleavage of caspase-3 was reduced by continuous activation of Akt (Figure 2D).

### α-Mangostin Induces Apoptosis by Targeting RXRα

**FIGURE 3:**
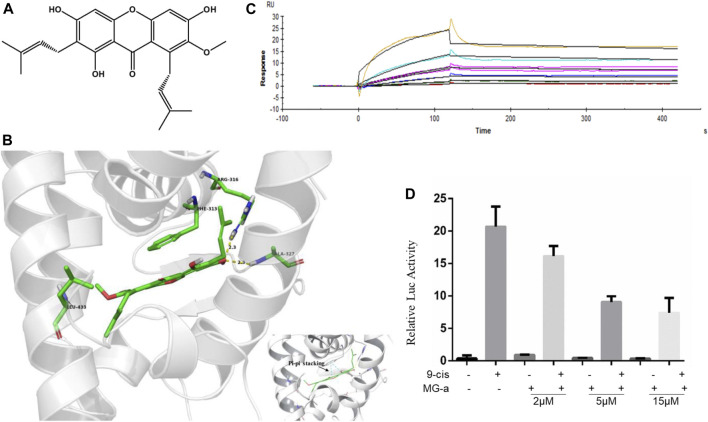
α-Mangostin induces apoptosis by targeting RXRα. **(A)** The structural formula of α-Mangostin. **(B)** The results of molecular docking: α-Mangostin interacts with amino acid residues Arg-316, Ala327, and Leu-433 of RXRα LBD. A pi-pi stacking is formed with amino acid residue Phe-313. **(C)** Antagonistic effect of α-Mangostin (2, 5, 15 μM). 293 T cells transfected with PBind-RXR/LBD and PG5 expression vector were treated with or without 10–7 mol/L 9-cisRA in the absence or presence of different concentrations of α-Mangostin. Reporter activities were measured and normalized. One of three independent experiments is shown. **(D)** SPR analysis of the interaction of MG-a with RXRα (α-Mangostin solution was injected into the RXR-LBD protein channel and blank channel at seven concentrations (0.625, 1.25, 2.5, 5, 10, 20, and 40 nM).

α-Mangostin belongs to the xanthone group of compounds (Chen et al., 2018). Its structural formula is shown in Figure 3A. In the current study, molecular docking was further examined to explore the binding of RXRα with α-Mangostin. The aligned structures of the original X-ray and docking are shown in Figure 3B. α-Mangostin mainly depends on the hydrogen bond and π-π interaction with RXRα amino acid residues. The value of the docking energy between RXRα and α-Mangostin is −10.235 kcal/mol. The results showed that the docking effect was good and indicated that α-Mangostin may have a strong binding effect on RXRα. The binding energy values are related to the interaction between the compounds and amino acid residues of target proteins.

RXRα is usually in an inactivated state as a transcription factor bound with a repressor. If ligands combine with RXRα and block the repressor, RXRα can be bound with the specific sequence in the target promoter and promote the expression of the target gene. The Dual-luciferase assays shows the results (Figure 3C) of the competitive binding of α-Mangostin to the RXRα ligand-binding domain. The natural ligand 9-cis-RA could significantly activate the reporting gene system, but this activity was inhibited by different concentrations of α-Mangostin (2, 5, and 15 μM).

The SPR measurements show that the gradient concentrations of α-Mangostin (0.625, 1.25, 2.5, 5, 10, 20, and 40 μM) flowed through the chip channel. As can be seen from the curve of α-Mangostin binding with RXRα (Figure 3D), the signal was obvious. The results showed that the KD value was 3.897*10^–5^ M and thus the binding affinity of RXRα for α-Mangostin was expecting. Moreover, the dissociation process between α-Mangostin and RXRα was slow and occurred via another process other than a common non-covalent combination, indicating that the binding of α-Mangostin and RXRα may be mainly covalent.

### α-Mangostin Induces the Degradation of RXRα/tRXR and Inhibits Akt Activity in MDA-MB-231 Cells

**FIGURE 4:**
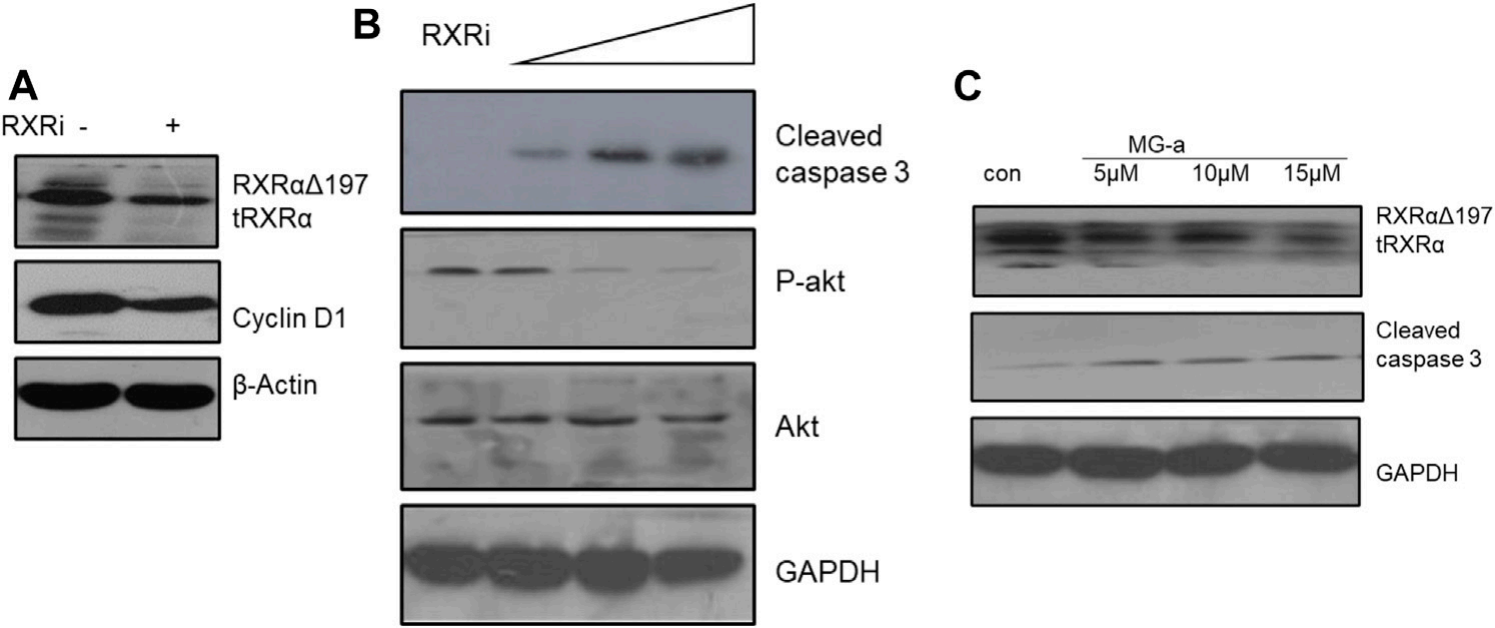
α-Mangostin induces the degradation of RXRα/tRXR and inhibits Akt activity in MDA-MB-231 cells. **(A,B)** The expression levels of cyclin D1 and P-Akt were determined by western blotting analysis in MDA-MB-231 cells after knockdown of RXRα. **(C)** The expression levels of RXRα/tRXR and cleaved caspase-3 were determined by western blotting analysis in MDA-MB-231 cells after α-Mangostin treatment.

Based on the above experiments, we demonstrated that α-Mangostin can target binding to RXRα/tRXRα and can also induce the apoptosis of breast cancer cells through the Akt pathway. However, it is unclear as to what role RXRα/tRXRα has in the regulation of PI3K/Akt signaling. To explore this, we used siRNA technology to knockdown RXRα/truncated RXRα (Figure 4A), and then detected the effect of α-Mangostin on Akt by western blotting (Figure 4B). The results show treatment with α-Mangostin suppressed the phosphorylation of Akt and activated caspase-3 after knockdown of RXRα/truncated RXRα (Figure 4C).

### The Effect of α-Mangostin on MDA-MB-231 Cell Cycle Depends on Cyclin D1

**FIGURE 5:**
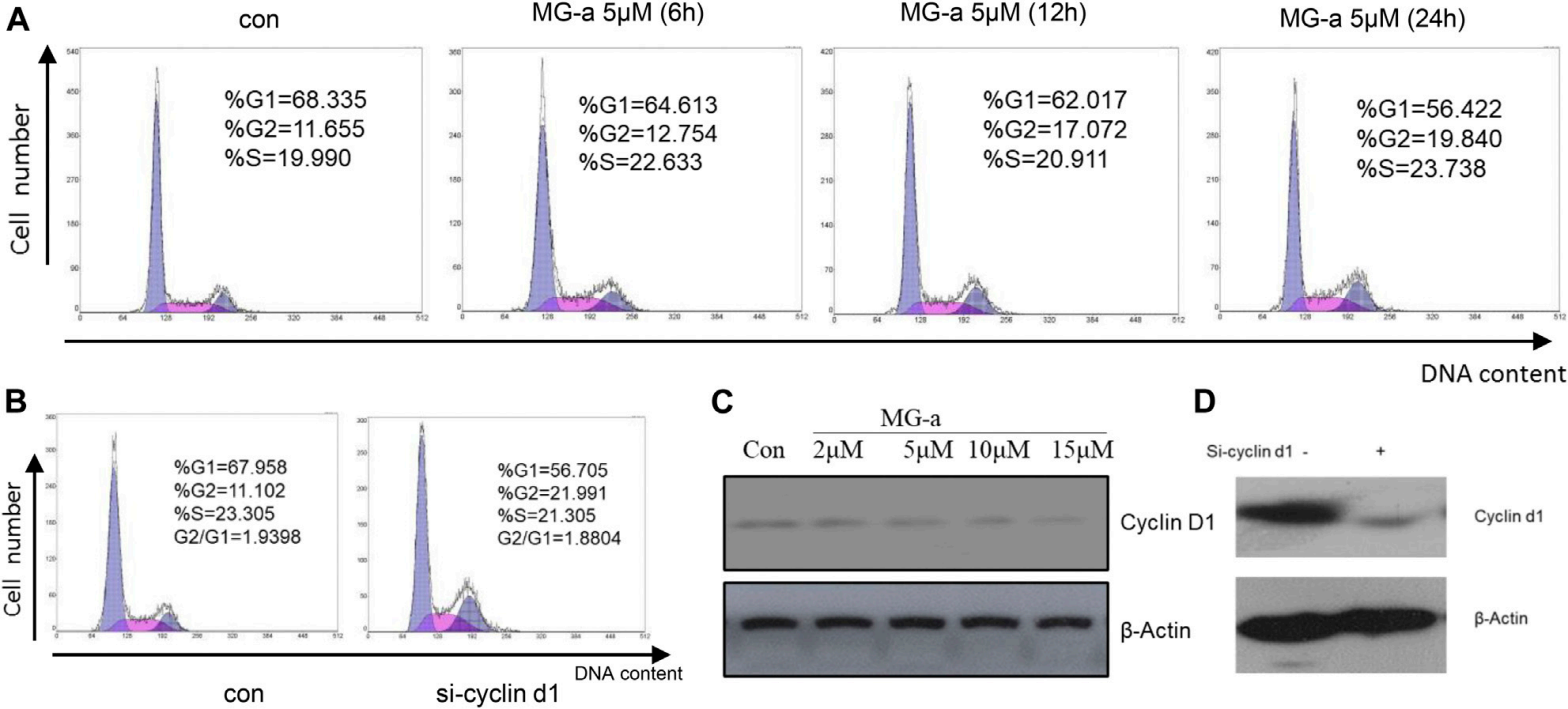
The effect of α-Mangostin on the MDA-MB-231 cell cycle depends on cyclin D1. **(A)** The sub-G1 ratio in MDA-MB-231 cells treated with MG-a was examined by flow cytometry. Cells were exposed to 5 μM MG-a at the indicated times, after which cells were harvested and stained with PI. FACS was used to measure the sub-G1 percentage. **(B)** The sub-G1 ratio in MDA-MB-231 cells treated with siCCND1 was examined using flow cytometry. **(C,D)** The expression levels of cyclin D1 were determined by western blotting analysis in MDA-MB-231 cells after α-Mangostin treatment **(C)** and knockdown of RXRα **(D)**.

Our studies have shown that α-Mangostin can significantly inhibit the proliferation of human breast cancer cells. We know that inhibition of cell proliferation is usually closely related to blockade of the cell cycle. Therefore, to comprehensively explore the molecular mechanism of the inhibitory effect of α-Mangostin, we analyzed its effect on the cell cycle of MDA-MB-231. In the flow cytometry analysis, it was found that the proportion of S phase cells increased significantly after treatment for 6 h with α-Mangostin (5 μM). and the proportion of cells in G2/M also increased after treatment for 12 h with α-Mangostin (5 μM). Meanwhile, the proportion of MDA-MB-231 cells in G0/G1 showed a decreasing trend after treatment with 5 μM α-Mangostin. This suggests that α-Mangostin can arrest MDA-MB-231 cells in S and G0/G1 phase (Figure 5A).

Cyclin D1 is a regulator of cell cycle protein-dependent kinase CDKs, which have an important effect on the blockade of the cell cycle (Body et al., 2017). The effects of siCCND1 on inhibiting MDA-MB-231 cell proliferation and blockade of the cell cycle (Figure 5B). We treated the MDA-MB-231 cells with a concentration gradient of α-Mangostin for 24 h, and then the expression of cyclin D1 was detected by western blotting. Cyclin D1 expression decreased with increased α-Mangostin concentration (Figure 5C). siRNA knockdown of RXRα resulted in obviously reduced expression of cyclin D1 (Figure 5D), indicating that RXRα was able to regulate cyclin D1. Therefore, we speculate that the blockade of the cell cycle by α-Mangostin depends on the RXRα/cyclin D1 signaling pathway.

### α-Mangostin Suppressed the Migration and Invasion of TNBC Cells *in Vitro*

**FIGURE 6:**
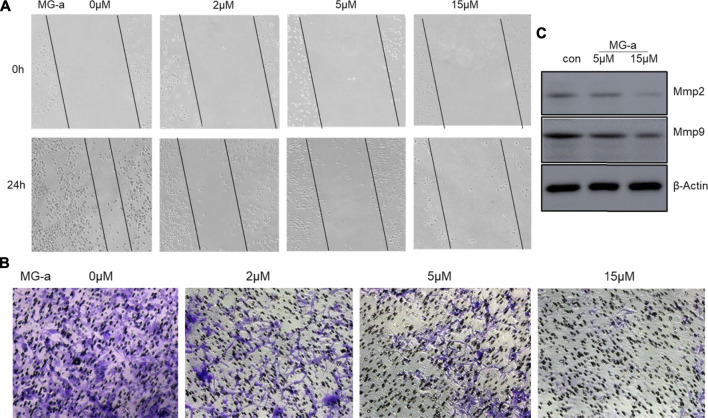
α-mangostin suppressed the migration and invasion of TNBC Cells *in Vitro*. **(A)** The cell migration ability was determined by wound healing assay. **(B)** The cell migration and invasion were determined by Transwell assay. **(C)** The expression levels of Mmp2 and Mmp9 were determined by western blotting analysis in MDA-MB-231 cells after α-Mangostin treatment.

To examine the anticancer effect of α-Mangostin on TNBC cells, we treated the highly aggressive MDA-MB-231 cells with α-Mangostin in different concentrations (2, 5, and 15 mM, respectively). Then we used wound-healing and transwell assay to determine whether α-Mangostin had the potential to inhibit breast cancer cell migration and invasion. α-Mangostin could concentration dependently slow down the wound healing process comparing to the control group (Figures 6A similar effect was observed in transwell assay, the invaded cells were decreased in α-Mangostin -treated groups when compared with the control group (Figure 6B). All these results suggested that α-Mangostin had the anticancer capability through influencing the proliferation, migration, and invasion of TNBC cells.

### The Inhibitory Effect of α-Mangostin on Breast Cancer Cell Migration and Invasion Is Also Dependent on the tRXR/Akt/Cyclin D1 Pathway

**FIGURE 7:**
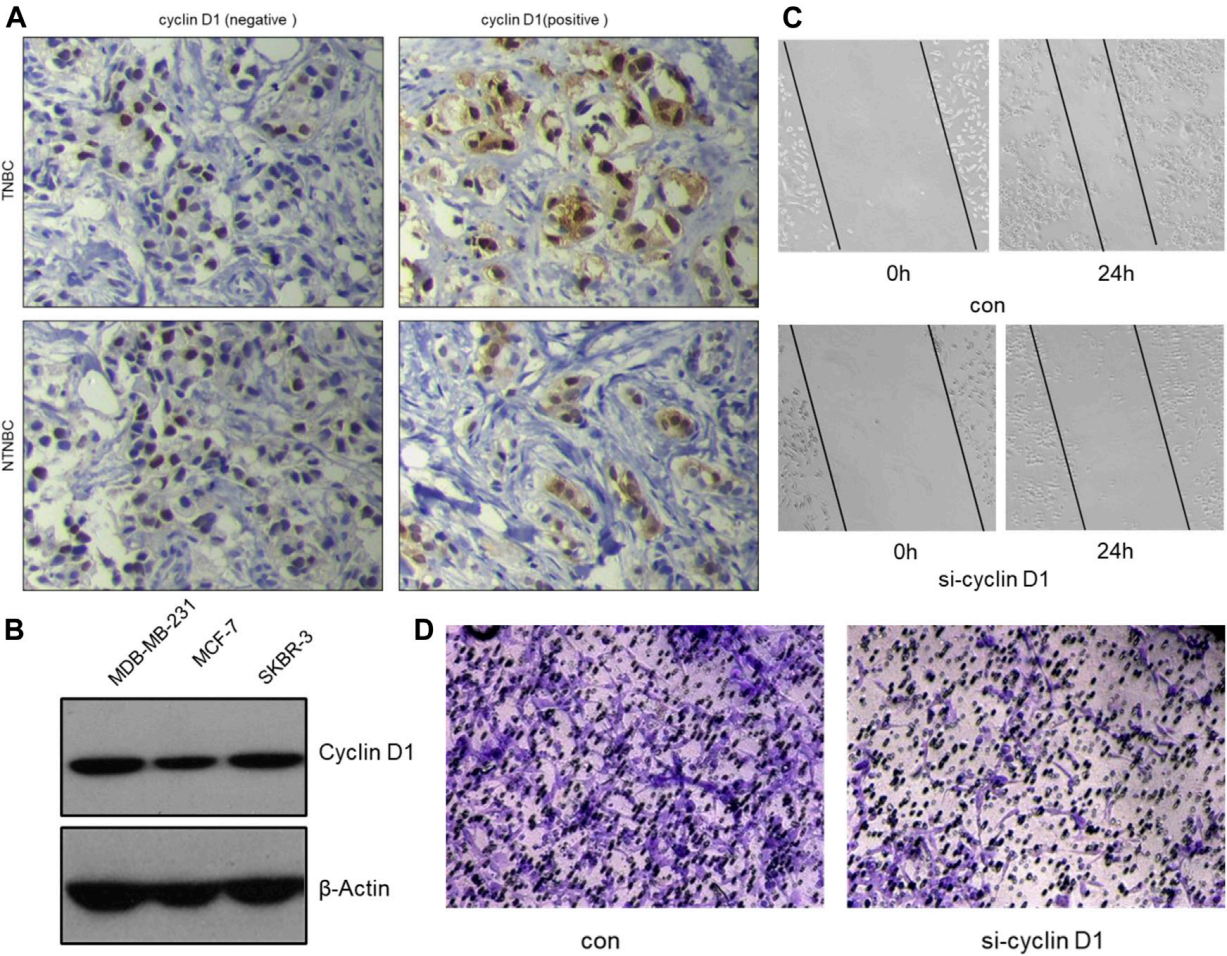
The inhibitory effect of α-mangostin on breast cancer cell migration and invasion is also dependent on the tRXR/Akt/cyclin D1 pathway. **(A)** Representative images for the different expression of cyclin D1 by immunohistochemistry in tissues samples from TNBC and NTNBC patients. **(B)** The different expression of cyclin D1 by western blotting in different breast cancer cell lines. **(C,D)** The inhibitory effect of MG-a on MDA-MB-231 cells migration/invasion.

Clinical studies show high expression of Cyclin D1 are often found in aggressive breast cancer, especially TNBC (Figure 7A). The clinical samples were collected from The First Affiliated Hospital of Gannan Medical University.) We speculate that cyclin D1 play a major role to promote tumor cell invasion and metastasis. Therefore, the MDA-MB-231 cells were used to verify the effect of knocking down Cyclin D1 on cell migration and invasion. The results of the Wound Healing Assay, the Transwell Assay and Western Blot Analysis all showed the migrated ability and invasive ability of MDA-MB-231 cells decline after being treated with si- Cyclin D1(Figure 7C,D).

## Discussion

The antitumor activity of α-Mangostin has been demonstrated in numerous studies (Asasutjarit et al., 2019; Ittiudomrak et al., 2019). Apoptosis of breast cancer cells induced by α-Mangostin has also been reported (Lee et al., 2010; Mardianingrum et al., 2020), but its specific molecular mechanism remains unclear. In this study, we first validated the findings of previous studies and verified that α-Mangostin could induce apoptosis of breast cancer cells *in vitro*. Flow cytometry experiments demonstrated that α-Mangostin promoted apoptosis with an efficiency that was similar to paclitaxel. Cell apoptosis is under the control of many signal transduction pathways and changes in homeostasis (Bissoli and Muscari, 2020). The cleavage of PARP proteins is a necessary factor for programmed cell death. α-Mangostin could induce PARP cleavage in breast cancer cells in a concentration and time-dependent manner. It also had a strong effect on inhibiting proliferation and inducing apoptosis in three breast cancer cell lines.

In many tumor cells including breast cancer cells, the nuclear receptor RXRα is hydrolyzed by a restricted protease. This results in RXRα with N-terminal deletion (Chen et al., 2014), known as tRXRα (truncated RXRα). Subcellular localization studies indicated that tRXRα is distributed in the cytoplasm. tRXRα can interact with p85 (a regulatory subunit of PI3K), and then activate Akt and the downstream PI3K/Akt pathway, promoting tumor processes (Xia et al., 2016). tRXRα is expressed specifically in tumor cells as an essential growth and survival factor. Thus, it is considered as a potential anti-tumor target. The ligand-binding domain of RXRα with N-terminal deletion remained the same as RXRα in our experiments. α-Mangostin can specifically target both RXRα and tRXRα, and we confirmed that α-Mangostin can degrade RXRα/tRXR in a concentration-dependent manner. Because tRXRα was the main factor activating PI3K/Akt in tumor cells, α-Mangostin could also inhibit the activity of Akt in a concentration-dependent manner.

The PI3K/Akt signaling pathway is closely related to tumor development. Inhibition of Akt activation may affect several downstream proteins, and can also induce apoptosis and suppress proliferation of tumor cells. α-Mangostin can regulate BCL-2 protein family members, and it can also activate caspase-3 and then trigger apoptosis in breast cancer cells. BCL-2 family proteins and caspase-3 protein are closely related to apoptosis. Further experiments revealed that the caspase-3 was no longer activated by α-Mangostin in MDA-MB-231 cells transfected with continuously activated Akt (CA-Akt). Apoptosis was reversed, and the proliferation of cells was almost unaffected. Furthermore, α-Mangostin had little effect on apoptosis following the treatment of MDA-MB-231 cells with LY294002 Akt inhibitor. So α-Mangostin-induced apoptosis of breast cancer cells depends on the PI3K/Akt signaling pathway.

The loss of control of cell cycle regulation, resulting in unlimited tumor cell proliferation, is an essential attribute of tumors. The mechanism of cell cycle regulation disorder is an important cause of cell proliferation dropout, which leads to cancer. Cyclin D1 is a critical cell cycle regulator and a candidate proto-oncogene, whose deregulation has been implicated in the pathogenesis of several cancer types (Montalto and De Amicis, 2020), including breast cancer. It regulates cell cycle progression by binding to CDK4 or CDK6, forming tight complexes. Thus, cyclin D1 has been an area of focus in cancer research. In this study, the effect of α-Mangostin on the cell cycle of MDA-MB-231 was analyzed by flow cytometry. The results showed that α-Mangostin could arrest MDA-MB-231 cells in S and G2/M phases. The alterations in MDA-MB-231 cell cycle progression by treatment with α-Mangostin are dependent on cyclin D1.

The experiments verify that α-Mangostin has evident inhibition effects of invasion and metastasis of MDA-MB-231 cells. Many previous studies have demonstrated that Mmp-2 and Mmp-9 are closely related to tumor invasion and metastasis (Lee et al., 2010). The two matrix metalloproteinases have been used as indicators of invasive and migratory capacity in many studies. α-Mangostin was shown to significantly down-regulate Mmp-2 and Mmp-9 in Western blot experiments, thereby providing for the ability to inhibit invasion and metastasis of MDA-MB-231 cells. Do the inhibitory effects of α-Mangostin also depend mainly on the tRXR/Akt/cyclin D1 pathway? α-Mangostin can down-regulate the expression of cyclin D1, and the lower expression of cyclin D1 have inhibition effects on migration and invasion of MDA-MB-231 cells *in vitro*. It suggests that the inhibitory effect of α-Mangostin on breast cancer cell migration and invasion is also dependent on the tRXR/Akt/cyclin D1 pathway.

## Conclusion

α-Mangostin occupies the binding pocket of RXRα and that the ligand antagonizes the effects of RXRα. α-Mangostin induces apoptosis of breast cancer cells through the PI3K/Akt signaling pathway by targeting RXRα, and cyclin D1 has involved in this process.

## Data Availability

The original contributions presented in the study are included in the article/supplementary material, further inquiries can be directed to the corresponding author.
